# The ALDH2 rs671 Polymorphism Affects Post-Stroke Epilepsy Susceptibility and Plasma 4-HNE Levels

**DOI:** 10.1371/journal.pone.0109634

**Published:** 2014-10-14

**Authors:** Heng Yang, Zhi Song, Guo-Ping Yang, Bi-Kui Zhang, Min Chen, Tian Wu, Ren Guo

**Affiliations:** 1 Department of Pharmacy, The Third Xiangya Hospital, Central South University, Changsha, Hunan, China; 2 Department of Neurology, The Third Xiangya Hospital, Central South University, Changsha, Hunan, China; 3 Department of Central Laboratory, The Third Xiangya Hospital, Central South University, Changsha, Hunan, China; Sungkyunkwan University, Republic of Korea

## Abstract

Recent studies have demonstrated the protective effect of mitochondrial aldehyde dehydrogenase 2 (ALDH2) in cardiovascular diseases. Increased levels of the potential ALDH2 substrate 4-hydroxynonenal (4-HNE) are involved in myocardial/cerebral ischemia accompanied by a high level of oxidative stress. In this investigation, we first performed a case-control study to explore the potential association of ALDH2 rs671 polymorphism and post-stroke epilepsy (PSE). Then, we performed an in vitro study to determine whether the overexpression of ALDH2 could decrease the level of oxidative stress and the apoptosis ratio induced by 4-HNE. There was a significant difference in the distribution of the allele and genotype frequencies of the rs671 polymorphism between PSE patients and ischemic stroke (IS) patients. Individuals with the rs671 A allele showed significantly higher levels of plasma 4-HNE. The overexpression of ALDH2 partially blocked the increased levels of malondialdehyde (MDA), reactive oxygen species (ROS) and apoptosis ratio induced by 4-HNE and also partially restored the ALDH2 activity in PC12 cells; these effects were reversed in the presence of εV1-2. Our results suggest that the ALDH2 rs671 polymorphism is associated with PSE susceptibility and affects the 4-HNE levels. Targeting ALDH2 might be a useful strategy for the treatment or prevention of PSE.

## Introduction

Epilepsy is the second most common disease of the nervous system only after cerebrovascular disease [1]. Epilepsy is caused by a variety of pathogenic factors and has been shown to be a chronic, recurrent, transient syndrome resulting from brain dysfunction. This condition is characterized by abnormal discharge in brain neurons, and the frequent attacks also cause progressive damage to brain neurons. There is evidence that stroke is one of the main epileptogenic factors in patients with secondary epilepsy, especially in the elderly [2], [3]. Post-stroke epilepsy has a negative effect on stroke prognosis and the quality of life. A Canadian research group reported that post-stroke epilepsy was associated with an increased utilization of resources, an increased length of hospital stay and a decrease in both the 30-day and 1-year survival rates [4]. The identification of a new method for predicting the occurrence of post-stroke epilepsy may result in the development of new strategies for the prevention and treatment of post-stroke epilepsy.

Oxidative stress is usually accepted as a vital factor that contributes to the cardiac and neuronal damage in ischemic injury [5], [6]. 4-hydroxy-2-nonenal (4-HNE), an α, β-unsaturated hydroxyalkenal produced in the process of lipid peroxidation, is generally considered to be a specific marker of oxidative stress. Previous studies have also shown that 4-HNE levels increased in genetic stroke-prone rats and rats with stroke experimentally induced by middle cerebral artery occlusion (MCAO) and that the increased 4-HNE was associated with aggravated ischemic brain damage[7], [8]. The underlying mechanism may be that 4-HNE disturbs the normal structure of key enzymes by forming protein adducts [9]. Mitochondrial aldehyde dehydrogenase 2 (ALDH2) is a key enzyme that metabolizes reactive aldehydes such as MDA, 4-HNE and 1-palmitoyl-2-oxovaleroyl phosphatidylcholine (POVPC) to acetic acid and also detoxifies reactive oxygen species (ROS)-generated aldehyde adducts to exert its cardioprotective effects [10].

ALDH2 is expressed in a large number of tissues, especially in the liver, kidney and muscle, it is also present in organs with abundant mitochondrial content, including the heart and brain [11]. The ALDH2 gene is mapped to chromosome 12 in the region of q24.2. A common single nucleotide polymorphism (SNP) in exon 12 of the ALDH2 gene leads to a genetic codon change in which glutamate is replaced by lysine at position 504 (Glu504Lys, also known as rs671 or ALDH2*2) [12], [13]. This functional SNP has been proven to have an adverse effect on the dehydrogenase activity of ALDH2, decreasing its normal activity by approximately 90%. Recent studies have shown that the Glu504Lys polymorphism may contribute to cardiovascular diseases. Guo et al. reported that the carriers of the ALDH2 rs671 A allele had an increased risk of coronary artery disease, possibly due to interfering HDL-C levels and endothelial ADMA levels [14]. Xu F et al. reported that rs671 could be considered a genetic risk marker for acute coronary syndrome [15]. Paradoxically, some studies have also reported that the ALDH2 rs671 polymorphism was unrelated to an increased risk of coronary heart disease and myocardial infarction [16], [17]. The ALDH2 rs671 polymorphism has the potential to define the risk factors and to individualize stroke treatment before the onset of epilepsy. However, the role of the rs671 polymorphism in post-stroke epilepsy has never been studied.

In the present study, we first conducted a case-control study to explore the potential association between the ALDH2 rs671 polymorphism and susceptibility to epilepsy. We further explored the beneficial effects of ALDH2 on 4-HNE induced injury in PC12 cells, because protein kinase C epsilon (PKCε) is reported to participate in ALDH2 activation [18], [19], [20], we therefore explored whether the antioxidant effect by overexpression of ALDH2 is also involved in PKCε signaling pathway.

## Materials and Methods

### Subjects

Our study recruited 225 post-stroke epilepsy (PSE) patients, 240 ischemic stroke (IS) patients and 267 healthy control subjects; all were enrolled in the outpatient clinics at The Third Xiangya Hospital in Hunan from September 2011 to June 2013. All patients and controls were Han Chinese who lived in Changsha or nearby counties. Furthermore, all subjects had no prior seizures before the stroke event and were matched for age and sex. IS was defined by focal neurological signs or symptoms of vascular origin that persisted longer than 24 h and was confirmed by brain CT and/or MRI using baseline conditions and brain CT using contrast medium after 48–72 h. PSE patients were defined based on their clinical symptoms and a positive EEG test. A 5-ml venous blood sample was drawn into an ethylene diamine tetraacetic acid-containing tube.

Written informed consent was obtained from all subjects. The study was performed with the approval of the Ethics Committee of the Third Xiangya Hospital of Central South University.

### Genotyping

Genomic DNA samples were extracted from peripheral blood leukocytes according to standard phenol/chloroform protocols. The ALDH2 rs671 polymorphism was genotyped using polymerase chain reaction-restriction fragment length polymorphism (PCR-RFLP) as described previously [21].

### Measurement of the 4-HNE concentration in the plasma

Peripheral 4-HNE often exists in the form of stable adducts. In this study, the level of HNE in protein adducts was measured using an ELISA kit (R&D, Minneapolis, USA) following the instructions provided by the manufacturer as described previously [22].

### Measurement of MDA levels

The MDA levels were determined using an MDA Detecting kit (WEIAO BioTech), according to the manufacturer's instructions.

### ALDH2 enzymatic activity

The ALDH2 activity was determined spectrophotometrically by monitoring the reductive reaction of NAD^+^ to NADH according to the manufacturer's instruction (GenMed Scientifics Inc., Wilmington, USA). The assays were measured at 25°C in a 1-ml reaction system containing 33 mM sodium pyrophosphate (pH 8.8), 0.8 mM NAD+, 15 µM propionaldehyde and 0.1 ml of the cell lysates. The production of NADH was determined spectrophotometrically by monitoring the change in absorbance intensity at 340 nm every 30 s for 5 min. The ALDH2 activity is expressed as µmol NADH/min/mg protein.

### Cell viability MTT assay

PC12 cells were plated in the 96-well plate at a density of 1×10^6^ cells/mL, each well was filled with 200 µL cell suspension. PBS was added around 96-well plate for sealing. Cells were incubated with 5% CO2 at 37°C. After synchronizing 12 h, added 100 µL medium containing corresponding concentration 4-HNE (0.05 mM, 0.1 mM and 0.15 mM) and incubated with 5% CO2 at 37°C. The 96-well plate was took out at 24 h, MTT was added to each well with a final concentration of 0.5 mg/mL, and then the plate was incubated with 5% CO2 at 37°C for another 4 h. Then samples were quantified spectroscopically at 560 nm using the spectrophotometer.

### Cell culture and transfection

PC12 cells were purchased from the Wuhan cell collection center and cultured in RPMI-1640 medium (Gibco) with 10% fetal bovine serum (Gibco) in a 5% CO_2_ incubator at 37°C. The ALDH2 gene was amplified by PCR and subcloned into the pIRES2-EGFP vector (Invitrogen). The control vectors or vectors carrying the ALDH2 gene were transfected into PC12 cell using Lipofectamine LTX with Plus (Invitrogen) according to the manufacturer's instructions. The cells were divided into 6 groups (n = 8 per group): (1) control group; (2) 4-HNE group, PC12 cells treated with 0.1 mM 4-HNE; (3) +ALDH2(O) group, PC12 cells overexpressing ALDH2 +0.1 mM 4-HNE; (4) +εV1-2 group, PC12 cell overexpressing ALDH2 +0.01 mM 4-HNE +10^−6^ M εV1–2; (5) +Vector group, PC12 transfected with vector +0.1 mM 4-HNE; and (6) +Vehicle, PC12 cells treated with 0.1 mM 4-HNE +0.1% DMSO.

### Annexin V-FITC and PI Staining Analysis

In order to assess apoptosis, PC12 cells were plated onto 6-well culture plates with different treatments. Following staining according to manufacturer's protocol, the apoptosis analysis of cell was performed by flow cytometry (FCM).

### Statistical analysis

SPSS software (Version 11.5) was used for the statistical analysis. The data were expressed as the mean ±SD. The differences in the genotype and allele frequencies between the groups were compared using a χ2 test. The differences among the groups were compared using a one-way ANOVA. *P*<0.05 was considered to be statistically significant.

## Results

### Baseline Characteristics

The demographic characteristics of all subjects in this study are shown in Table 1. The baseline characteristics were not significantly different between the IS patients and the PSE patients.

**Table 1:** General characteristics of the IS and PSE patients.

Parameter	IS (n = 240)	PSE(n = 225)	*P*
Gender (male/female)	142/98	137/88	NS
Age, year	61.15±8.77	63.27±7.56	NS
BMI, kg/m^2^	23.66±3.02	23.35±2.73	NS
SBP, mmHg	143±13	146±11	NS
DBP, mmHg	87±9	85±8	NS
Cr, µmol/L	86.33±10.55	87.29±15.33	NS
HDL-C, mmol/L	1.34±0.35	1.29±0.48	NS
LDL-C, mmol/L	2.66±0.47	2.59±0.63	NS
TG, mmol/L	1.96±0.57	1.92±0.93	NS
TC, mmol/L	4.85±0.76	4.80±0.74	NS

Cr: Creatinine; TG: Triglyceride; TC: Total cholesterol.

### Distribution of allele and genotype frequencies of the rs671 polymorphism

To test the possible association between ALDH2 rs671 polymorphism and PSE susceptibility, we compared the distribution of rs671 in IS patients with/without epilepsy. The genotype and frequencies of the ALDH2 rs671 polymorphism in the study are shown in table 2. The distribution of the genotypes in both study groups complied with Hardy-Weinberg equilibrium. The comparison of the genotype distribution between the IS patients and the PSE patients demonstrated that the frequency of the rs671 A allele in the PSE patients was significantly higher than that in the IS patients (31.8% *vs* 21.3%, *P* = 0.00036, OR = 1.98, 95%CI = 1.36–2.87). When the groups were stratified according to history of alcohol consumption, a more significant difference in the frequency of the rs671 A allele was found in the subjects with a negative history of alcohol consumption (37.5% *vs* 24.3%, *P* = 0.00010, OR = 2.37, 95%CI = 1.54–3.65).

**Table 2:** Frequencies of ALDH2 genotypes and alleles in IS patients and PSE patients.

Genotype	IS	PSE	OR^a^
**Overall subjects**	**(n = 240)**	**(n = 225)**	
GG	156 (65.0%)	109 (48.4%)	1.00 (reference)
GA	66 (27.5%)	89 (39.6%)	1.98 (1.36∼2.87)^a^
AA	18 (7.5%)	27 (12%)	
**Negative history of drinking**			
GG	106 (59.9%)	65 (38.7%)	1.00 (reference)
GA	56 (31.6%)	80 (47.6%)	2.37 (1.54∼3.65)^b^
AA	15 (8.5%)	23 (13.7%)	
**Positive history of drinking**			
GG	50 (79.3%)	44 (77.2%)	1.00 (reference)
GA	10 (15.9%)	9 (15.8%)	1.14 (0.48∼2.71)
AA	3 (4.8%)	4 (7.0%)	

a Adjusted for age, sex, hypertension, BMI, and drinking.

b *P* = 0.00036,

c *P* = 0.00010.

### Plasma 4-HNE concentration in subjects

**Figure 1:**
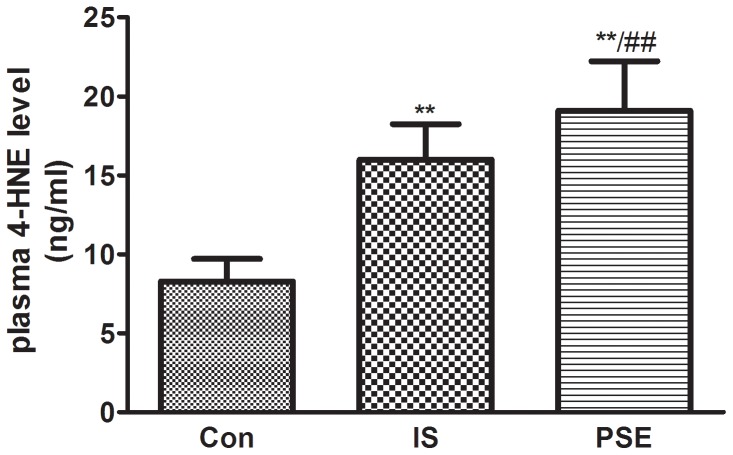
Plasma 4-HNE concentrations were higher in IS and PSE patients. The descriptive results are expressed as the mean ±SD, n = 180. Con: Healthy controls; IS: Ischemic stroke patients; PSE: Post-stroke epilepsy patients. **P<0.01, compared with healthy controls, #*P*<0.05, compared with IS patients.

Previous studies have shown that increased 4-HNE levels are involved in the process of myocardial/cerebral ischemia accompanied by a high level of oxidative stress. So in this study, we randomly selected 180 subjects in IS patients, PSE patients and healthy control subjects (matched for age and sex) respectively, to observed the difference of 4-HNE level, we found that plasma 4-HNE concentrations were significantly higher in stroke patients and PSE patients compared with the healthy controls (Fig 1). Furthermore, PSE patients showed the highest levels of 4-HNE.

### The effect of the ALDH2 rs671 polymorphism on 4-HNE concentrations

**Figure 2:**
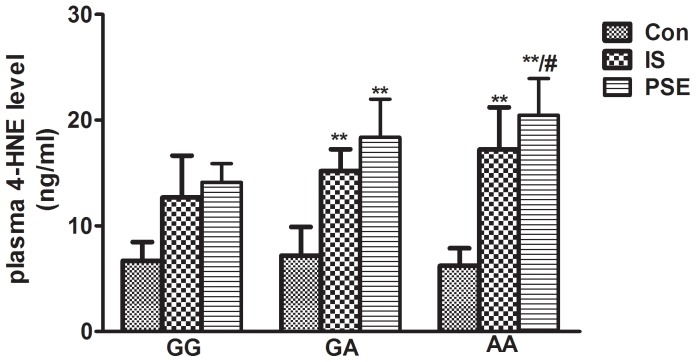
IS patients and PSE patients with the rs671 A allele showed increased 4-HNE levels. The descriptive results are expressed as the mean ±SD, n = 120. GG: GG genotype; GA: GA genotype: AA: AA genotype. **P<0.01, compared with corresponding GG genotype, #*P*<0.05, compared with corresponding GA genotype.

Reactive aldehydes, including 4-HNE and MDA, are potential substrates of ALDH2. To explore the potential relationship between the rs671 polymorphism and plasma 4-HNE concentrations, we separated the subjects (healthy controls, IS patients, PSE patients) into 3 subgroups (n = 120, randomly selected in each subgroup) according to their genotypes and compared the 4-HNE concentrations between them. The results are presented in Fig 2. A significant increase in 4-HNE levels was observed in individuals with an rs671 A allele both in IS patients and PSE patients. There was no significant difference in 4-HNE concentrations between the genotypes in the healthy controls (Fig 2).

### Cell survival under different concentration of 4-HNE

**Figure 3:**
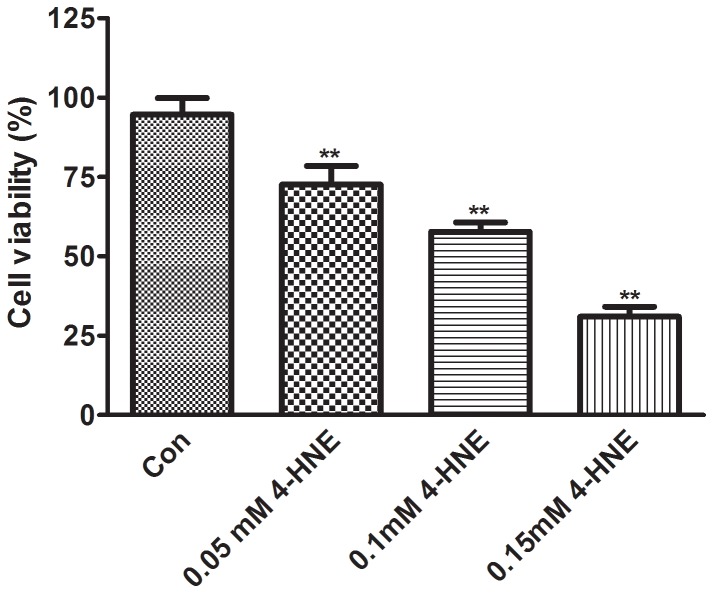
Cell survival under different concentration of 4-HNE. The descriptive results of cell viability are expressed as the mean ±SD, n = 6. Con: Control group; 0.05 mM 4-HNE: 0.05 mM 4-HNE treated group; 0.1 mM 4-HNE: 0.1 mM 4-HNE treated group; 0.15 mM 4-HNE: 0.15 mM 4-HNE treated group. **P<0.01, compared with Con.

We performed a MTT assay to determine the ideal concentration for PC12 cells damaged by 4-HNE after 24 h. The cell viability rate was significantly decreased with the increase of 4-HNE concentration. As shown in Fig 3, the corresponding cell viabilities of PC12 cells were 72.58%±5.83%, 57.67%±2.96%, 30.95%±3.15% at the concentration of 0.05 mM, 0.1 mM, 0.15 mM 4-HNE, respectively. Based upon this result, we used 0.1 mM 4-HNE to induce the cell damage.

### Overexpression of ALDH2 decreased oxidative stress induced by 4-HNE in PC12 cells

**Figure 4:**
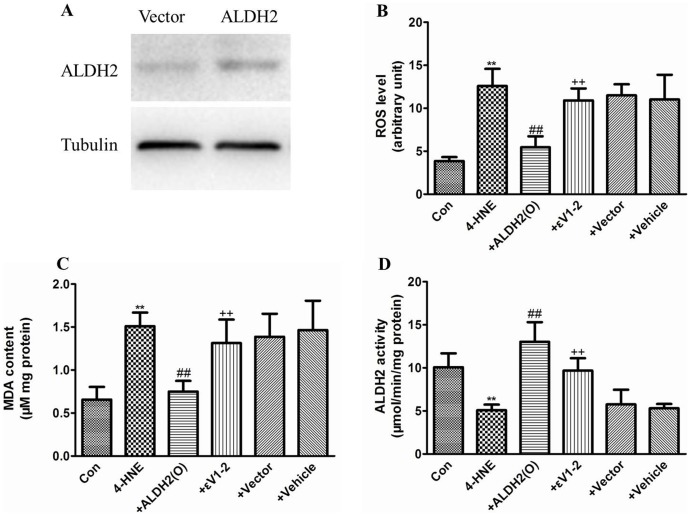
Overexpression of ALDH2 protected PC12 cells from 4-HNE-induced injury. (A) A representative image of an immunoblot showing the overexpression of ALDH2 in PC12 cells. (B) The ROS levels in PC12 cells for each group (n = 8); (C) The MDA levels in PC12 cells for each group (n = 8); (D) The ALDH2 activity for each group (n = 8). All values are expressed as the mean ±SD. Con: Control group; 4-HNE: 4-HNE treated group; +ALDH2(O): 4-HNE+ALDH2 overexpression; +εV1-2: 4-HNE+ALDH2 overexpression+εV1–2; +Vector: 4-HNE+Vector; and +Vehicle: 4-HNE+DMSO. **P<0.01, compared with Con, ##*P*<0.01, compared with 4-HNE, ++ *P*<0.01, compared with +ALDH2(O).

Increased 4-HNE levels are found in ischemic brains and are generally recognized as a marker for oxidative stress. Individuals with rs671 A allele showed a decreased activity of ALDH2, in order to test the association between ALDH2 activity and 4-HNE induced cell injury, we overexpressed ALDH2 in PC12 cells to determine whether the overexpression of ALDH2 can decrease the level of oxidative stress induced by 4-HNE. As shown in Fig 4, treatment of PC12 cells with 0.1 mM 4-HNE for 24 h significantly increased the MDA level accompanied by an increased level of ROS in PC12 cells, and as expected, the ALDH2 activity in PC12 cells was also significantly depressed. The overexpression of ALDH2 partially blocked the increased levels of MDA and ROS induced by 4-HNE and also partially restored the ALDH2 activity. However, these beneficial effects were reversed in the presence of εV1–2 (a specific inhibitor of PKC-ε). Compared to the 4-HNE group, the vehicle and vector treatments did not show any significant effect on cellular ROS, MDA and ALDH2 activity.

### Overexpression of ALDH2 rescued PC12 cells from apoptosis induced by 4-HNE

**Figure 5:**
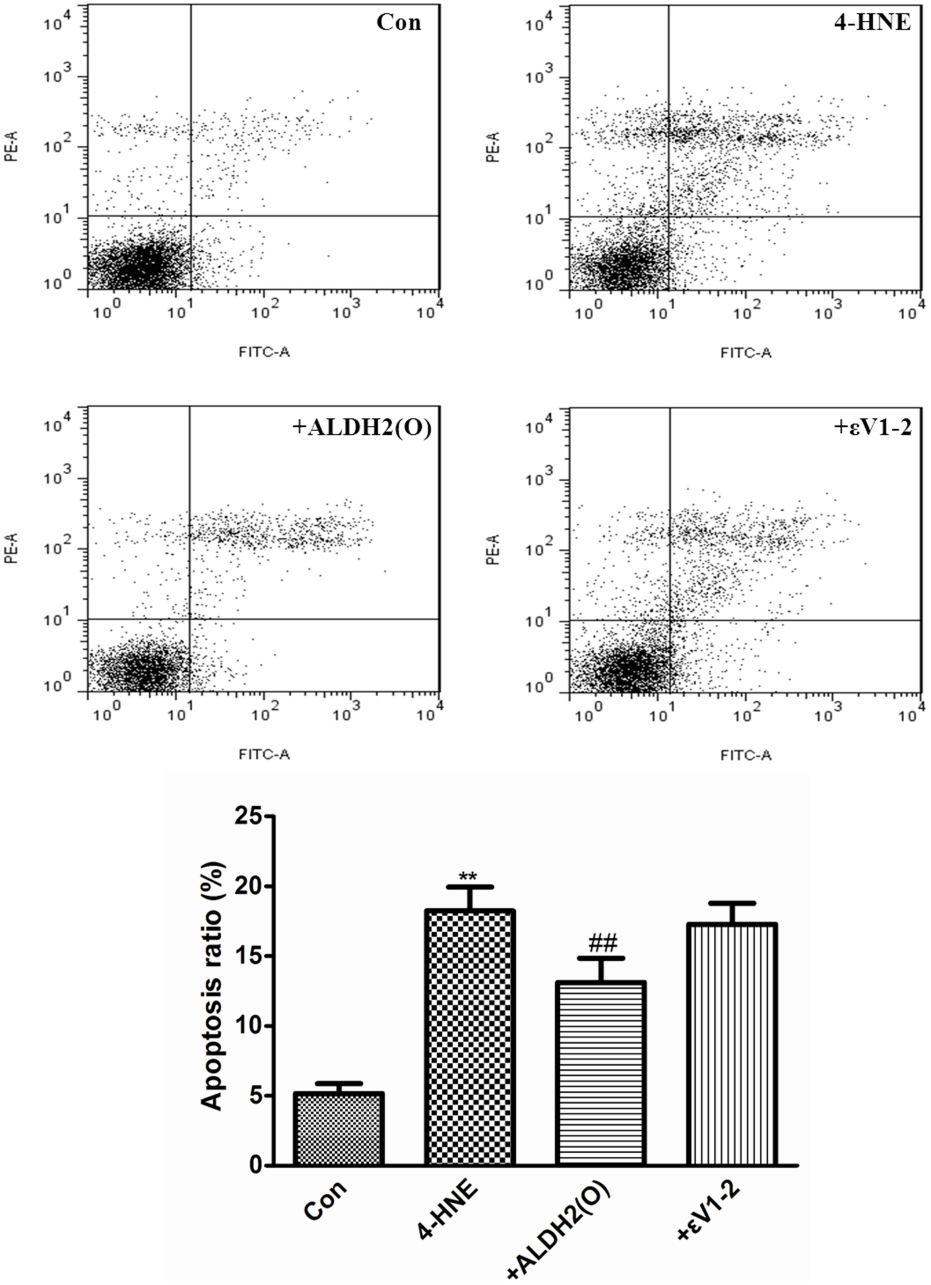
Overexpression of ALDH2 rescued PC12 cells from apoptosis induced by 4-HNE. The descriptive results of apoptosis ratio are expressed as the mean ±SD, n = 3. Con: Control group; 4-HNE: 4-HNE treated group; +ALDH2(O): 4-HNE+ALDH2 overexpression; +εV1–2: 4-HNE+ALDH2 overexpression+εV1–2. **P<0.01, compared with Con, ##*P*<0.01, compared with 4-HNE.

FCM stained with annexin V-FITC/PI was used to detect the apoptosis of the PC12 cells induced by 4-HNE. AS shown in Fig 5, treatment of PC12 cells with 0.1 mM 4-HNE for 24 h significantly increased the apoptosis ratio. When compared with 4-HNE group, the overexpression of ALDH2 partially decreased the apoptosis ratio (13.10%±1.73% *vs* 18.22%±1.71%, *P*<0.01). However, pretreatment with εV1-2 reversed this beneficial effect.

## Discussion

Using a case-control study, we demonstrated that the functional SNP rs671 of ALDH2 is associated with post-stroke epilepsy. We also found significantly increased 4-HNE levels in stroke patients and post-stroke epilepsy patients. Due to the relationship between ALDH2 and 4-HNE, we further evaluated the effect of the rs671 polymorphism on 4-HNE levels. As expected, the carriers of the ALDH2*2 allele showed significantly increased 4-HNE levels. In cultured PC12 cells, the overexpression of ALDH2 decreased the level of oxidative stress and apoptosis induced by 4-HNE; this effect was partially blocked by an inhibitor of PKCε.

To our knowledge, this is the first report describing the association between the ALDH2 rs671 polymorphism and PSE susceptibility. ALDH2 was first associated with the flush that occurs on the face when a person consumes alcohol and alcohol addiction [23], [24]. In recent years, many studies have demonstrated the role of ALDH2 in the biological metabolism of nitrates. Nitroglycerin can be catalyzed into 1, 2–2 glycerin nitrate and nitrite in the presence of ALDH2, which is critical for the vasodilation effect of nitroglycerin [25], [26]. Recently, the cardiovascular-protective effects of ALDH2 were reported by several groups. The restoration of ALDH2 alleviated hypoxia–reoxygenation-induced cardiac dysfunction [22], possibly by an autophagy-dependent pathway [27]. Similar protection from ALDH2 was reported by Guo et al. in stroke-prone spontaneously hypertensive rats [7]. A common functional SNP in exon 12 of ALDH2 leads to a dramatically decreased enzymatic activity [28]. This polymorphism has been identified as a risk factor for myocardial infarction, lacunar infarcts and Alzheimer [29], [30], [31]. Our data provide the first evidence that the rs671 polymorphism is associated with PSE susceptibility; individuals with ALDH2*2 showed an increased susceptibility to PSE. Because ALDH2 plays a role in alcohol consumption and moderate ethanol administration is beneficial for protecting against stroke [32], we divided the subjects into two subgroups according to their history of alcohol consumption. The presence of ALDH2*2 significantly increased the risk of PSE in subjects without a history of alcohol consumption, suggesting that alcohol consumption may have an effect on the development of cardiovascular diseases. Moderate alcohol intake might prevent cardiovascular diseases by activating ALDH2 [7].

4-HNE and MDA are representative reactive aldehydes produced during the process of lipid peroxidation, and they both play an important role in cardiovascular diseases. High 4-HNE levels were found in stroke-prone spontaneously hypertensive rats and stroke patients [7], [8], and a similar elevation of 4-HNE was also observed in ischemic hearts from rats [22]. Therefore, we examined the plasma levels of 4-HNE in our subjects. As expected, higher levels of 4-HNE were found in stroke patients and PSE patients than in healthy controls, and the PSE group showed the highest levels of 4-HNE. Our results found a positive correlation between 4-HNE and PSE. Many reactive aldehydes, including 4-HNE and MDA, are potential substrates of ALDH2. Due to the relationship between 4-HNE and ALDH2, we first explored the effect of the rs671 polymorphism on plasma 4-HNE levels in PSE patients. Our results provide the first evidence that the carriers of ALDH2*2 have increased 4-HNE levels after stroke with or without PSE. Given that ALDH2 can barely be detected in the blood of patients, the plasma levels of 4-HNE and the presence of the ALDH2 rs671 polymorphism might be two feasible and reliable indexes to evaluate stroke patients for PSE susceptibility. Because there are elevated levels of 4-HNE in the ischemic brain, we then performed an in vitro study to investigate the effect of 4-HNE on PC12 cells. Our data show that 4-HNE significantly decreased ALDH2 activity and increased the levels of ROS, MDA and apoptosis ratio. The overexpression of ALDH2 partially restored the ALDH2 activity and lowered the level of oxidative stress and apoptosis, this effect was blocked by the presence of PKCε inhibitors. 4-HNE is widely recognized as a specific marker of oxidative stress. The high levels of ROS induced by 4-HNE might promote thiol oxidation in the active center of ALDH2, which in turn inhibits the activity of ALDH2. In agreement with previous studies, our study also identified PKCε as a regulator upstream of ALDH2.

Current clinical strategies for PSE are aimed at controlling the seizures with medication. However, the treatments show little benefit for the prognosis of PSE. Our study may provide a new strategy to prevent PSE. Either the ALDH2 rs671 polymorphism or plasma levels of 4-HNE could be considered to be predictors for PSE. Because individuals with ALDH2*2 have a high level of 4-HNE and face an increased risk of PSE, activators of ALDH2 have therapeutic potential as new preventive remedies for PSE.

In summary, the results of our study show for the first time a significant association between the ALDH2 rs671 polymorphism and an increased risk of PSE. In stroke patients with or without PSE, the ALDH2*2 of rs671 was associated with higher levels of plasma 4-HNE. 4-HNE administration induced increased levels of oxidative stress and apoptosis, 4-HNE also depressed ALDH2 activity in PC12 cells. The overexpression of ALDH2 could partially alleviate the impairments through a PKCε-dependent pathway. Therefore, in stroke patients, especially in individuals with the ALDH2*2 allele, ALDH2 activation could be a useful strategy to treat or to prevent PSE. Further studies are needed to explore the exact role of ALDH2 in the process of epilepsy; for example, the association between ALDH2 and ATP-sensitive potassium channels warrants further study.

## References

[1] Scharfman HE (2007) The neurobiology of epilepsy. Curr Neurol Neurosci Rep 7: 348–354. 17618543 10.1007/s11910-007-0053-z PMC2492886

[2] Forsgren L, Bucht G, Eriksson S, Bergmark L (1996) Incidence and clinical characterization of unprovoked seizures in adults: a prospective population-based study. Epilepsia 37: 224–229. 8598179 10.1111/j.1528-1157.1996.tb00017.x

[3] Li X, Breteler MM, de Bruyne MC, Meinardi H, Hauser WA, et al (1997) Vascular determinants of epilepsy: the Rotterdam Study. Epilepsia 38: 1216–1220. 9579923 10.1111/j.1528-1157.1997.tb01219.x

[4] Burneo JG, Fang J, Saposnik G (2010) Impact of seizures on morbidity and mortality after stroke: a Canadian multi-centre cohort study. Eur J Neurol 17: 52–58. 10.1111/j.1468-1331.2009.02739.x 19686350

[5] Cherubini A, Polidori MC, Bregnocchi M, Pezzuto S, Cecchetti R, et al (2000) Antioxidant profile and early outcome in stroke patients. Stroke 31: 2295–2300. 11022053 10.1161/01.str.31.10.2295

[6] Li SY, Li Q, Shen JJ, Dong F, Sigmon VK, et al (2006) Attenuation of acetaldehyde-induced cell injury by overexpression of aldehyde dehydrogenase-2 (ALDH2) transgene in human cardiac myocytes: role of MAP kinase signaling. J Mol Cell Cardiol 40: 283–294. 16403513 10.1016/j.yjmcc.2005.11.006

[7] Guo JM, Liu AJ, Zang P, Dong WZ, Ying L, et al (2013) ALDH2 protects against stroke by clearing 4-HNE. Cell Res 23: 915–930. 23689279 10.1038/cr.2013.69 PMC3698638

[8] Lee WC, Wong HY, Chai YY, Shi CW, Amino N, et al (2012) Lipid peroxidation dysregulation in ischemic stroke: plasma 4-HNE as a potential biomarker. Biochem Biophys Res Commun 425: 842–847. 22898049 10.1016/j.bbrc.2012.08.002

[9] Renner A, Sagstetter MR, Harms H, Lange V, Gotz ME, et al (2005) Formation of 4-hydroxy-2-nonenal protein adducts in the ischemic rat heart after transplantation. J Heart Lung Transplant 24: 730–736. 15949734 10.1016/j.healun.2004.02.021

[10] Lagranha CJ, Deschamps A, Aponte A, Steenbergen C, Murphy E (2010) Sex differences in the phosphorylation of mitochondrial proteins result in reduced production of reactive oxygen species and cardioprotection in females. Circ Res 106: 1681–1691. 20413785 10.1161/CIRCRESAHA.109.213645 PMC3127199

[11] Stewart MJ, Malek K, Crabb DW (1996) Distribution of messenger RNAs for aldehyde dehydrogenase 1, aldehyde dehydrogenase 2, and aldehyde dehydrogenase 5 in human tissues. J Investig Med 44: 42–46. 8689400

[12] Crabb DW, Edenberg HJ, Bosron WF, Li TK (1989) Genotypes for aldehyde dehydrogenase deficiency and alcohol sensitivity. The inactive ALDH2(2) allele is dominant. J Clin Invest 83: 314–316. 2562960 10.1172/JCI113875 PMC303676

[13] Yoshida A, Huang IY, Ikawa M (1984) Molecular abnormality of an inactive aldehyde dehydrogenase variant commonly found in Orientals. Proc Natl Acad Sci U S A 81: 258–261. 6582480 10.1073/pnas.81.1.258 PMC344651

[14] Guo YJ, Chen L, Bai YP, Li L, Sun J, et al (2010) The ALDH2 Glu504Lys polymorphism is associated with coronary artery disease in Han Chinese: Relation with endothelial ADMA levels. Atherosclerosis 211: 545–550. 20417517 10.1016/j.atherosclerosis.2010.03.030

[15] Xu F, Chen YG, Xue L, Li RJ, Zhang H, et al (2011) Role of aldehyde dehydrogenase 2 Glu504lys polymorphism in acute coronary syndrome. J Cell Mol Med 15: 1955–1962. 21958412 10.1111/j.1582-4934.2010.01181.x PMC3918050

[16] Hashimoto Y, Nakayama T, Futamura A, Omura M, Nakarai H, et al (2002) Relationship between genetic polymorphisms of alcohol-metabolizing enzymes and changes in risk factors for coronary heart disease associated with alcohol consumption. Clin Chem 48: 1043–1048. 12089173

[17] Nakamura Y, Amamoto K, Tamaki S, Okamura T, Tsujita Y, et al (2002) Genetic variation in aldehyde dehydrogenase 2 and the effect of alcohol consumption on cholesterol levels. Atherosclerosis 164: 171–177. 12119207 10.1016/s0021-9150(02)00059-x

[18] Budas GR, Disatnik MH, Chen CH, Mochly-Rosen D (2010) Activation of aldehyde dehydrogenase 2 (ALDH2) confers cardioprotection in protein kinase C epsilon (PKCvarepsilon) knockout mice. J Mol Cell Cardiol 48: 757–764. 19913552 10.1016/j.yjmcc.2009.10.030 PMC2837767

[19] Churchill EN, Disatnik MH, Mochly-Rosen D (2009) Time-dependent and ethanol-induced cardiac protection from ischemia mediated by mitochondrial translocation of varepsilonPKC and activation of aldehyde dehydrogenase 2. J Mol Cell Cardiol 46: 278–284. 18983847 10.1016/j.yjmcc.2008.09.713 PMC2675554

[20] Chen CH, Budas GR, Churchill EN, Disatnik MH, Hurley TD, et al (2008) Activation of aldehyde dehydrogenase-2 reduces ischemic damage to the heart. Science 321: 1493–1495. 18787169 10.1126/science.1158554 PMC2741612

[21] Wang RS, Nakajima T, Kawamoto T, Honma T (2002) Effects of aldehyde dehydrogenase-2 genetic polymorphisms on metabolism of structurally different aldehydes in human liver. Drug Metab Dispos 30: 69–73. 11744614 10.1124/dmd.30.1.69

[22] He L, Liu B, Dai Z, Zhang HF, Zhang YS, et al (2012) Alpha lipoic acid protects heart against myocardial ischemia-reperfusion injury through a mechanism involving aldehyde dehydrogenase 2 activation. Eur J Pharmacol 678: 32–38. 22266491 10.1016/j.ejphar.2011.12.042

[23] Bosron WF, Lumeng L, Li TK (1988) Genetic polymorphism of enzymes of alcohol metabolism and susceptibility to alcoholic liver disease. Mol Aspects Med 10: 147–158. 3067025 10.1016/0098-2997(88)90019-2

[24] Ferguson RA, Goldberg DM (1997) Genetic markers of alcohol abuse. Clin Chim Acta 257: 199–250. 9118563 10.1016/s0009-8981(96)06444-3

[25] Chen Z, Foster MW, Zhang J, Mao L, Rockman HA, et al (2005) An essential role for mitochondrial aldehyde dehydrogenase in nitroglycerin bioactivation. Proc Natl Acad Sci U S A 102: 12159–12164. 16103363 10.1073/pnas.0503723102 PMC1189320

[26] Mackenzie IS, Maki-Petaja KM, McEniery CM, Bao YP, Wallace SM, et al (2005) Aldehyde dehydrogenase 2 plays a role in the bioactivation of nitroglycerin in humans. Arterioscler Thromb Vasc Biol 25: 1891–1895. 16051882 10.1161/01.ATV.0000179599.71086.89

[27] Ma H, Guo R, Yu L, Zhang Y, Ren J (2011) Aldehyde dehydrogenase 2 (ALDH2) rescues myocardial ischaemia/reperfusion injury: role of autophagy paradox and toxic aldehyde. Eur Heart J 32: 1025–1038. 20705694 10.1093/eurheartj/ehq253 PMC3076664

[28] Li Y, Zhang D, Jin W, Shao C, Yan P, et al (2006) Mitochondrial aldehyde dehydrogenase-2 (ALDH2) Glu504Lys polymorphism contributes to the variation in efficacy of sublingual nitroglycerin. J Clin Invest 116: 506–511. 16440063 10.1172/JCI26564 PMC1351000

[29] Nagasawa H, Wada M, Arawaka S, Kawanami T, Kurita K, et al (2007) A polymorphism of the aldehyde dehydrogenase 2 gene is a risk factor for multiple lacunar infarcts in Japanese men: the Takahata Study. Eur J Neurol 14: 428–434. 17388993 10.1111/j.1468-1331.2007.01700.x

[30] Takagi S, Iwai N, Yamauchi R, Kojima S, Yasuno S, et al (2002) Aldehyde dehydrogenase 2 gene is a risk factor for myocardial infarction in Japanese men. Hypertens Res 25: 677–681. 12452318 10.1291/hypres.25.677

[31] Zhou S, Huriletemuer, Wang J, Zhang C, Zhao S, et al (2010) Absence of association on aldehyde dehydrogenase 2 (ALDH2) polymorphism with Mongolian Alzheimer patients. Neurosci Lett 468: 312–315. 19914339 10.1016/j.neulet.2009.11.022

[32] Patra J, Taylor B, Irving H, Roerecke M, Baliunas D, et al (2010) Alcohol consumption and the risk of morbidity and mortality for different stroke types–a systematic review and meta-analysis. BMC Public Health 10: 258. 20482788 10.1186/1471-2458-10-258 PMC2888740

